# Docosahexaenoic Acid Suppresses Silica-Induced Inflammasome Activation and IL-1 Cytokine Release by Interfering With Priming Signal

**DOI:** 10.3389/fimmu.2019.02130

**Published:** 2019-09-20

**Authors:** Kathryn A. Wierenga, Josephine Wee, Kristen N. Gilley, Lichchavi D. Rajasinghe, Melissa A. Bates, Mikhail A. Gavrilin, Andrij Holian, James J. Pestka

**Affiliations:** ^1^Department of Biochemistry and Molecular Biology, Michigan State University, East Lansing, MI, United States; ^2^Institute for Integrative Toxicology, Michigan State University, East Lansing, MI, United States; ^3^Department of Food Science and Human Nutrition, Michigan State University, East Lansing, MI, United States; ^4^Division of Pulmonary, Critical Care and Sleep Medicine, Ohio State University, Columbus, OH, United States; ^5^Department of Biomedical and Pharmaceutical Sciences, Center for Environmental Health Sciences, University of Montana, Missoula, MT, United States; ^6^Department of Microbiology and Molecular Genetics, Michigan State University, East Lansing, MI, United States

**Keywords:** macrophage, cell death, inflammasome, silica, omega-3 fatty acids, *in vitro*

## Abstract

Occupational exposure to respirable crystalline silica (cSiO_2_) has been etiologically linked to human autoimmunity. Intranasal instillation with cSiO_2_ triggers profuse inflammation in the lung and onset of autoimmunity in lupus-prone mice; however, dietary supplementation with the omega-3 polyunsaturated fatty acid docosahexaenoic acid (DHA) abrogates these responses. Inflammasome activation, IL-1 cytokine release, and death in alveolar macrophages following cSiO_2_ exposure are early and critical events that likely contribute to triggering premature autoimmune pathogenesis by this particle. Here we tested the hypothesis that DHA suppresses cSiO_2_-induced NLRP3 inflammasome activation, IL-1 cytokine release, and cell death in the macrophage. The model used was the murine macrophage RAW 264.7 cell line stably transfected with the inflammasome adapter protein ASC (RAW-ASC). Following priming with LPS, both the canonical activator nigericin and cSiO_2_ elicited robust inflammasome activation in RAW-ASC cells, as reflected by IL-1β release and caspase-1 activation. These responses were greatly diminished or absent in wild-type RAW cells. In contrast to IL-1β, cSiO_2_ induced IL-1α release in both RAW-ASC and to a lesser extent in RAW-WT cells after LPS priming. cSiO_2_-driven effects in RAW-ASC cells were confirmed in bone-marrow derived macrophages. Pre-incubating RAW-ASC cells with 10 and 25 μM DHA for 24 h enriched this fatty acid in the phospholipids by 15- and 25-fold, respectively, at the expense of oleic acid. DHA pre-incubation suppressed inflammasome activation and release of IL-1β and IL-1α by nigericin, cSiO_2_, and two other crystals – monosodium urate and alum. DHA's suppressive effects were linked to inhibition of LPS-induced *Nlrp3, Il1b*, and *Il1a* transcription, potentially through the activation of PPARγ. Finally, nigericin-induced death was inflammasome-dependent, indicative of pyroptosis, and could be inhibited by DHA pretreatment. In contrast, cSiO_2_-induced death was inflammasome-independent and not inhibited by DHA. Taken together, these findings indicate that DHA suppresses cSiO_2_-induced inflammasome activation and IL-1 cytokine release in macrophages by acting at the level of priming, but was not protective against cSiO_2_-induced cell death.

## Introduction

Occupational exposure to airborne crystalline silica (cSiO_2_) has been linked to the prevalence of autoimmune disease (1). We have previously demonstrated that cSiO_2_ triggers the early onset and progression of systemic autoimmunity and glomerulonephritis in lupus-prone female NZBWF1 mice (2). In this model, intranasal instillation with cSiO_2_ induces profuse inflammation in the lung characterized by cytokine and chemokine secretion, lymphocyte infiltration, and autoantigen release. Collectively, these processes promote the development of pulmonary ectopic lymphoid structures (ELS) that drive autoimmune pathogenesis. Remarkably, supplementing the NZBWF1 mouse diets with the omega-3 polyunsaturated fatty acid (ω-3 PUFA) docosahexaenoic acid (C22:6 ω-3; DHA), a widely used dietary supplement extracted from cold-water fish, blocks cSiO_2_-triggered inflammation, ectopic lymphoid neogenesis, systemic autoimmunity, and nephritis (3, 4). Accordingly, supplementation with DHA and other ω-3 PUFAs may be an effective intervention against triggering of lupus onset, flaring, and/or progression by environmental agents such as cSiO_2_. However, the mechanisms behind DHA's suppression of cSiO_2_-accelerated pulmonary and systemic autoimmunity are unclear.

Phagocytosis by alveolar macrophages (AMΦs) is a primary line of defense against respirable particles. After cSiO_2_ is phagocytosed, it induces lysosomal membrane permeabilization that in turn elicits NLRP3 inflammasome oligomerization and caspase-1 activation (5–8). Caspase-1 selectively cleaves pro-IL-1β to mature IL-1β, and induces cell death via pyroptosis (9–11). The latter results in release of inflammatory mediators, alarmins, autoantigens, and reemergence of the cSiO_2_ particles into the alveolar space. Continuous repetition of this sequence promotes recruitment and activation of additional leukocytes in the lung, culminating in chronic inflammation and autoimmunity (5). Other crystals, including monosodium urate (MSU) (12), alum (8), and cholesterol (13), have also been shown to activate the NLRP3 inflammasome.

NLRP3 inflammasome activation requires a priming signal such as the pathogen-associated lipopolysaccharide (LPS), which upregulates expression of inflammasome components and IL-1 cytokines (14). Importantly, cytokines (e.g., IL-1β, TNF-α, and IL-6) and endogenous danger signals such as alarmins can similarly elicit this priming effect. The canonical alarmin IL-1α is constitutively expressed in myriad cell populations, including macrophages under steady state conditions, but its expression can be upregulated by proinflammatory or stress-associated stimuli (15). Rabolli et al. (16) reported that macrophages are a main source of IL-1α in the lung and that cSiO_2_ can induce release of this cytokine. Like IL-1β, IL-1α binds the IL-1R1 receptor on AMΦ, consequently activating NF-κB-driven expression of inflammasome proteins (15). Release of IL-1β and IL-1α in concert with the sustained presence of cSiO_2_ (17) then allows a feed-forward loop of inflammasome activation and pyroptotic cell death within MΦs that is capable of perpetually activating inflammation and autoimmune pathogenesis.

Previous studies suggest that DHA and other ω-3 PUFAs potentially interfere with cSiO_2_-induced NLRP3 inflammasome activation, release of IL-1 cytokines, and death of AMΦs (18–22). However, elucidation of how ω-3 PUFAs influence these responses is inherently difficult due to low numbers of AMΦs isolatable from animals or humans. The murine RAW 264.7 (RAW) cell line is a widely used MΦ model that has been cited in mechanism studies nearly 10,000 times since its discovery in 1977 by Raschke et al. (23). Importantly, the wild-type RAW (RAW-WT) cells lack the inflammasome adapter ASC (apoptosis-associated speck-like protein containing a CARD domain) that is crucial for NLRP3 inflammasome assembly (24). This can be rectified by transfection with the ASC gene thereby rendering this cell line capable of mounting an inflammasome response similar to primary AMΦs (25). Herein, we employed RAW-ASC and RAW-WT cells with and without inflammasome priming to test the hypothesis that DHA suppresses cSiO_2_-induced NLRP3 inflammasome activation, IL-1 cytokine release, and cell death in the macrophage.

## Materials and Methods

### RAW-WT and RAW-ASC MΦ Models

Murine-derived wild-type RAW 264.7 (RAW-WT) cells were purchased from the American Type Culture Collection (ATCC® TIB-71™). RAW-ASC cells were obtained by transfection with a fusion CFP-ASC protein. The open-reading frame of ASC was amplified from cDNA by PCR and inserted at the C-terminus of cyan fluorescent protein (CFP) of pLenti-CFP plasmid generated on the basis of pLenti6/V5 (Invitrogen Life Technologies, Carlsbad, CA) resulting in a fusion CFP-ASC protein, as described previously (26). Plasmid containing the fusion CFP-ASC protein (designated pLenti-CFP-ASC) was verified by Sanger sequencing. The lentiviral system was generated in the packaging cell line HEK293-FT (Invitrogen Life Technologies) and transfected with pLenti-CFP-ASC and helper plasmids pCMVΔR8.2 and pMD.G using Lipofectamine 2000 (Invitrogen Life Technologies). Cell culture medium containing virus was harvested at 48 and 72 h post-transfection, filtered, and concentrated at 3,200 x g for 30 min in Centricon C-20 columns, 100,000 MWCO (Millipore Sigma, Burlington, MA) resulting in a titer of 1–2E7 TU/ml. To restore inflammasome function, RAW 264.7 lacking expression of endogenous ASC were transduced with CFP-ASC lentivirus at 2–5 multiplicity of infection (MOI) in the presence of 6 μg/ml polybrene. After lentiviral transduction, 10–15% cells expressed fluorescent protein. Stably transduced cells were then selected with 5 μg/ml of blasticidin (InvivoGen) for 10 days, resulting in 90% fluorescent cells as observed by fluorescent microscopy. Both cell types were cultured in phenol red-free RPMI 1640 medium (Thermo Fisher Scientific, Waltham, MA) supplemented with 10% FBS (Thermo Fisher Scientific) and 1% Penicillin-streptomycin (Invitrogen Life Technologies) and sub-cultured every 2–4 days (24, 27).

### Preparation of Bone Marrow-Derived Macrophages (BMDMs)

All experimental protocols involving animals were reviewed and approved by the Institutional Animal Care and Use Committee at Michigan State University in accordance with the National Institutes of Health guidelines (AUF # PROTO201800113). Femurs were removed from 8 to 14 week old C57BL/6J mice and marrow was flushed from the bone with ice cold PBS. Cells were dissociated by pipetting and filtered through a 40 μm cell strainer. Cells were pelleted and resuspended in 1 mL red blood cell lysis buffer (Thermo Fisher Scientific) and incubated at room temperature for 10 min. An additional 10 mL PBS were added and cells were pelleted, counted, and plated at 5 × 10^6^ cells per 100 mm petri dish in DMEM (Thermo Fisher Scientific) supplemented with 10% FBS, 1% Penicillin-streptomycin, and 20% L929 supernatant as previously described (28). Medium was refreshed every 2–3 days. Adherent macrophages were used in experiments at 7 days after isolation.

### cSiO_2_ and Other Crystals

cSiO_2_ (Min-U-Sil-5, Pennsylvania Glass Sand Corp, Pittsburgh, PA) was prepared as previously described (29). Briefly, cSiO_2_ was suspended in 1 M HCl and washed for 1 h at 100°C. Following cooling, cSiO_2_ was washed 3 times with sterile water and dried at 200°C overnight. For treatments, acid washed cSiO_2_ was suspended in fresh, sterile Dulbecco's phosphate-buffered saline (DPBS, pH 7.4). Similarly, MSU crystals (InvivoGen) were suspended in DPBS at 5 mg/mL per the manufacturer's instructions. A 20 mg/mL stock suspension of alum crystals (InvivoGen) was prepared in sterile water and diluted 1:4 with PBS before use. All crystal suspensions were stored at 4°C for no longer than 2 weeks. Prior to use, crystal suspensions were vortexed thoroughly, sonicated for 1 min and added dropwise to wells to achieve the desired concentrations.

### DHA Preparation

DHA-bovine serum albumin (BSA) complexes were used to supplement cell culture media at physiologically relevant doses. The complexes were prepared as previously described (30, 31). Briefly, a 15% solution of fatty acid-free, endotoxin-, and fatty acid-free BSA (Millipore Sigma) was prepared in serum-free RPMI. Stocks of DHA (Cayman Chemical, Ann Arbor, MI) were diluted in EtOH to 11.76 mg/mL. A volume of the DHA corresponding to 20 mg DHA was transferred to glass tube and EtOH evaporated under a steady stream of N_2_. DHA was then dissolved in 4 mL of 0.05 M Na_2_CO_3_ for a final concentration of 5 mg/mL. The tube was flushed with N_2_ gas, vortexed, and incubated at room temperature for 1 h. DHA in Na_2_CO_3_ and 15% w/v BSA in RPMI were added to serum-free RPMI to obtain a final concentration of 2.5 mM DHA and 0.833 mM BSA (3:1 molar ratio). The tube was flushed with N_2_ and gently mixed for 30 min. The DHA-BSA complex solution was filter sterilized and aliquoted. Complexes were capped with N_2_ and stored at −20°C for no longer than 3 months.

### Experimental Design

**Figure 1:**
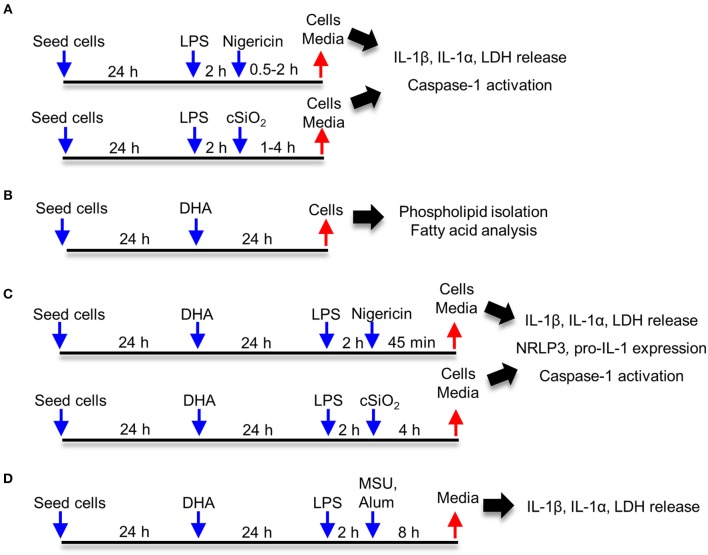
Experimental design. Studies focused on **(A)** characterization of cSiO_2_- and nigericin-induced inflammasome activation, IL-1 cytokine release and cell death in RAW-WT and RAW-ASC macrophages, **(B)** assessing effects of DHA supplementation on the fatty acid profile of membrane phospholipids, **(C)** determining how DHA impacts inflammasome activation, IL-1 cytokine release and cell death, and **(D)** assessing how DHA impacts inflammasome activation by monosodium urate (MSU) and alum crystals.

Approaches used for this study are summarized in Figure 1. Frequently used reagents can be found in Table S1. For comparison of RAW-WT or RAW-ASC MΦs, cells were plated at 3 × 10^5^ cells/well in 12 well plates, 1.5 × 10^5^ cells/well in 24 well plates, or 1.7 × 10^4^ cells/well in 96 well plates in order to achieve 70–90% confluency at the time of treatment. Unless otherwise noted, cells were cultured for 24 h in complete RPMI (phenol red-free RPMI 1640, 10% FBS, 1% penicillin-streptomycin), washed once with sterile DPBS and primed for 2 h with 20 ng/mL LPS in serum deprived RPMI (phenol red-free RPMI 1640, 0.25% FBS, 1% penicillin-streptomycin). Following priming with LPS (from *Salmonella enterica* serotype typhimurium containing <1% protein impurities, Millipore Sigma), cSiO_2_, nigericin (Millipore Sigma), MSU, alum, or vehicle (DPBS for cSiO_2_ and <0.3% EtOH for nigericin) was added to cultures dropwise. Cells were incubated with nigericin for 30 to 120 min, with cSiO_2_ for 1 to 4 h, and with MSU or alum for 8 h. Culture supernatants were collected for cytokine ELISAs and lactate dehydrogenase (LDH) assays, and cells were collected for RNA and protein extraction. For DHA supplementation studies, cells were seeded at 2.5 × 10^5^ cells/well in 12 well plates or 1.25 × 10^5^ cells/well in 24 well plates, as established in prior experiments to achieve 70–90% confluency at the time of treatment. Cells were grown 24 h in complete RPMI. Wells were then washed once with DPBS and media was replaced with RPMI containing 0.25% FBS and 10 or 25 μM DHA as a 3:1 complex with BSA (30, 32). Non-supplemented media (0 μM DHA) containing BSA was used as a vehicle control. After 24 h, wells were washed with DPBS and subjected to treatments as described above.

### IL-1 Cytokine Analyses

IL-1β and IL-1α release was measured using the mouse IL-1β/IL-1F2 DuoSet® ELISA and mouse IL-1α/IL-1F1 DuoSet® ELISA (R&D Systems, Minneapolis, MN) per the manufacturer's instructions.

### Caspase-1 Activity

Caspase-1 activation was determined in LPS-primed and unprimed RAW-ASC cells using the FAM-FLICA *in vitro* Caspase-1 kit (ImmunoChemistry Technologies, LLC, Bloomington, MN). This kit employs the fluorescent inhibitor probe FAM-YVAD-FMK to label active caspase-1 enzyme in living cells. To investigate the effect of cSiO_2_ on caspase-1 activation in RAW-ASC cells, 1.5 x 10^5^ cells per well were seeded in complete RPMI medium in a clear bottomed, black walled 24-well plate. After 24 h, cells were subjected to the treatments described above, with the exception that 15 μL of FAM-FLICA reagent (20x) was added 2 h before the end of incubation. Following treatment, plates were spun at 100 x g for 2 min to recover detached cells. Cells were gently washed with the provided wash buffer and centrifuged at 100 x g for 2 min three times. Cells were imaged with the EVOS FL Auto Cell Imaging System (Invitrogen Life Technologies) with 40x objective using the GFP light cube. For multi-well analysis, total green fluorescence intensity was measured at 492 ex and 520 em using surface scan mode of EnSpire™ Multilabel Plate Reader (PerkinElmer Inc., Waltham, MA). Total fluorescence intensity was normalized to total amount of protein as measured by Pierce™ BCA Protein Assay Kit (Thermo Fisher Scientific). Results from two separate experiments were combined and expressed as fold change relative to treatment control.

### Cell Death

At the conclusion of the nigericin or cSiO_2_ treatment periods, cell death was assessed by release of lactate dehydrogenase (LDH) as previously described (33). Briefly, 10% Triton X-100 (Millipore Sigma) was added to control wells designated for quantification of maximum kill (MK) at 2% (v/v) to induce maximum cell lysis. Media was collected from MK and sample wells and 50 μL media from each well added to an untreated, flat-bottomed 96-well plate. Serum-deprived RPMI was used as the sample blank and serum-deprived RPMI with 10% Triton-X was used as the MK blank. LDH reagent solution was prepared fresh as described (33) and 100 μL added to each well. Plates were incubated in the dark at room temperature for 15 min and read on a FilterMax F3 Multimode plate reader (Molecular Devices, San Jose, CA) at an absorbance wavelength of 492 nm. Cytotoxicity of samples was calculated as follows: 100%^*^[(sample_abs_ - sample blank_abs_)/(MK_abs_ - MK blank_abs_)]. Remaining cell culture medium was stored at −20°C until cytokine analysis.

### PPARγ Transcription Factor Assay

Samples were prepared using a Nuclear Extraction Kit (Active Motif Inc., Carlsbad, CA) per the manufacturer's instructions. Protein content of the nuclear extracts was quantified by Pierce™ BCA Protein Assay Kit (Thermo Fisher Scientific). PPARγ activity in nuclear extracts was assessed using the TransAM® PPARγ Transcription Factor ELISA kit (Active Motif Inc). Activity was expressed as fold-change relative to control.

### qRT-PCR

Following 6 h incubation with 20 ng/mL LPS, RNA was extracted using RNeasy Mini spin columns provided with the RNeasy Mini Kit (Qiagen, Germantown, MD). Following extraction, reverse transcription was performed using a High Capacity RNA to cDNA Reverse Transcription Kit (Invitrogen Life Technologies). Quantitative real-time qPCR was performed using specific Taqman probes for selected genes involved in NLRP3 inflammasome formation (*Nlrp3* Assay ID Mm00840904_m1, *Il1b* Assay ID Mm01336189_m1, *Il1a* Assay ID Mm00439620_m1, *Casp1* Assay ID Mm004438023_m1; Thermo Fisher Scientific) on the Applied Biosystems™ QuantStudio™ 7 real-time PCR system. Data were analyzed with Applied Biosystems™ Thermo Fisher Cloud using the RQ software and the relative quantification method. *Gapdh* (Assay ID Mm99999915_g1; Thermo Fisher Scientific) was used as the housekeeping gene. Relative copy number (RCN) for each gene was normalized to expression of *Gapdh* and calculated as described previously (34).

### SDS-PAGE and Western Blot

RAW-ASC macrophages were lysed in RIPA buffer containing Halt Protease Inhibitor (RIPA-HPI; Thermo Fisher Scientific). To assess IL-1α in supernatant, protein was concentrated by precipitation with methanol and chloroform (35). Briefly, 600 μL of methanol:chloroform (4:1) was added to 600 μL media supernatant. Samples were centrifuged at 12,000 x g for 5 min and the upper methanol layer removed. Methanol (600 μL) was added, samples were vortexed and centrifuged again. Supernatant was removed, and the pellet was dried under a gentle stream of N_2_. The pellet was then resuspended in 60 μL RIPA-HPI.

For assessment of NF-κB activation, nuclear and cytoplasmic extracts were prepared using a NE-PER Nuclear and Cytoplasmic Extraction Kit (Thermo Fisher Scientific) supplemented with Halt Protease Inhibitor according to the manufacturer's instructions and as previously described (36).

Protein lysates and supernatant concentrates were diluted in Laemmli sample buffer containing 5% β-mercaptoethanol and separated on a 4–20% gradient gel (Bio-Rad Laboratories, Hercules, CA) with 1x TGS buffer (Bio-Rad Laboratories). Protein was transferred to a Trans-Blot® Turbo™ RTA Mini low fluorescent PVDF membrane (Bio-Rad Laboratories) using Trans-Blot® Turbo™ gel transfer stacks (Bio-Rad Laboratories) and the Trans-Blot® Turbo™ Transfer Device (Bio-Rad Laboratories) as directed by the manufacturer. Efficient protein transfer was confirmed by staining membranes with a solution containing Coomassie Brilliant Blue R-250 Dye. The membranes were placed in an iBind Western Device (Thermo Fisher Scientific) and the primary and secondary antibody solutions and washes were added to the corresponding chambers. Dilutions of antibodies were made with the iBind Fluorescent Detection Solution Kit (Thermo Fisher Scientific) according to the manufacturer's protocol. The primary antibodies, namely goat anti-IL-1β (R&D Systems), rabbit anti-IL-1α (Cell Signaling Technology, Danvers, MA), rabbit anti-caspase-1 (p20) (Adipogen, San Diego, CA), mouse anti-IκBα (Cell Signaling Technology), rabbit anti-p-IKKα/β (Cell Signaling Technology), and rabbit anti-NF-κB p65 (Cell Signaling Technology), were diluted at 1:1000 for cell lysates or 1:500 for supernatant and placed into the primary antibody chamber. Rabbit anti-GAPDH (Cell Signaling Technology) and rabbit anti-beta-actin (Cell Signaling Technology) were diluted 1:1000 and 1:2000, respectively, and used for normalization of proteins detected in the lysate or cytoplasmic fraction. Mouse anti-PCNA (Cell Signaling Technology) was diluted 1:1000 and used for normalization of proteins detected in the nuclear fraction. Similarly, the secondary antibodies (donkey anti-goat IRDye 800CW, goat anti-mouse 800CW, goat anti-rabbit 680RD) from LI-COR Biosciences System (LI-COR Biosciences, Lincoln, NE, USA) were diluted at 1:3000 and placed into their corresponding chamber. The membranes were left in the iBind system for >2.5 h at room temperature before scanning with the LI-COR Odyssey Infrared Imaging System (LI-COR Biosciences).

Data shown are mean percent of total FA ± SEM (*n* = 8 per group) as determined by GLC. FA levels significantly different from control (0 μM DHA) represented by asterisks (^*^p < 0.05, ^**^p < 0.01).

### Fatty Acid Analysis

Cells were seeded at a density of 1.5 × 10^6^ cells per 100 mm dish. After 24 h in complete RPMI, wells were washed once with DPBS and media switched to serum-deprived RPMI supplemented with 25 μM DHA as described above. After 24 h, cells were washed once with DPBS, collected in ice-cold DPBS, and pelleted at 1,000 x g for 3 min. Cell pellets were resuspended in 1.4 mL ice-cold DPBS as previously described (37). Each sample was divided in half and stored in 1.6 mL screw-cap tubes in order to perform the phospholipid isolation in technical duplicates. Samples were then snap frozen and stored at −80°C until further analysis. Total lipids were extracted according to the method by Bligh and Dyer (38) and phospholipids were isolated by solid phase extraction as described previously (39). Isolated phospholipids were stored at −80°C in a 3:1 solution of hexane/isopropanol until methylation. The exogenous heptadecanoic acid, C17:0 (NuChek Prep, Elysian, MN), was added to isolated phospholipids as an internal standard. Samples were methylated using methanolic BF_3_ (Millipore Sigma) as previously described (40). FAMEs were analyzed by gas chromatography using a Shimadzu GC equipped with a flame ionization detector. Samples were separated on a J&W DB-23 30 m capillary column (Agilent, Santa Clara, CA) with an inner diameter of 0.25 μm and a flow rate of 0.82 mL/min, with helium as the carrier gas. The injection temperature and detector temperature were 250°C and the column temperature ranged from 120 to 240°C. A 16-standard FAME mix (NuChek Prep) was used to identify peaks of interest.

### Statistical Analyses

Student's *t*-tests were used to compare two groups when applicable. If groups were determined non-parametric or determined to have unequal variance by the Shapiro-Wilk test for normality or the *F*-test for equal variance, respectively, they were analyzed using the Mann-Whitney U test. Comparison of multiple groups was accomplished by one-way ANOVA, and comparison of individual groups was accomplished using Tukey's test. If groups were determined non-parametric or determined to have unequal variance by the Shapiro-Wilk test for normality or the F-test equal variance, respectively, they were analyzed by the Kruskal-Wallis test. In this case, *post hoc* comparison of individual groups was accomplished using Dunn's test.

## Results

### Nigericin- and cSiO_2_- Induced IL-1β Release Is LPS- and ASC-Dependent

**Figure 2:**
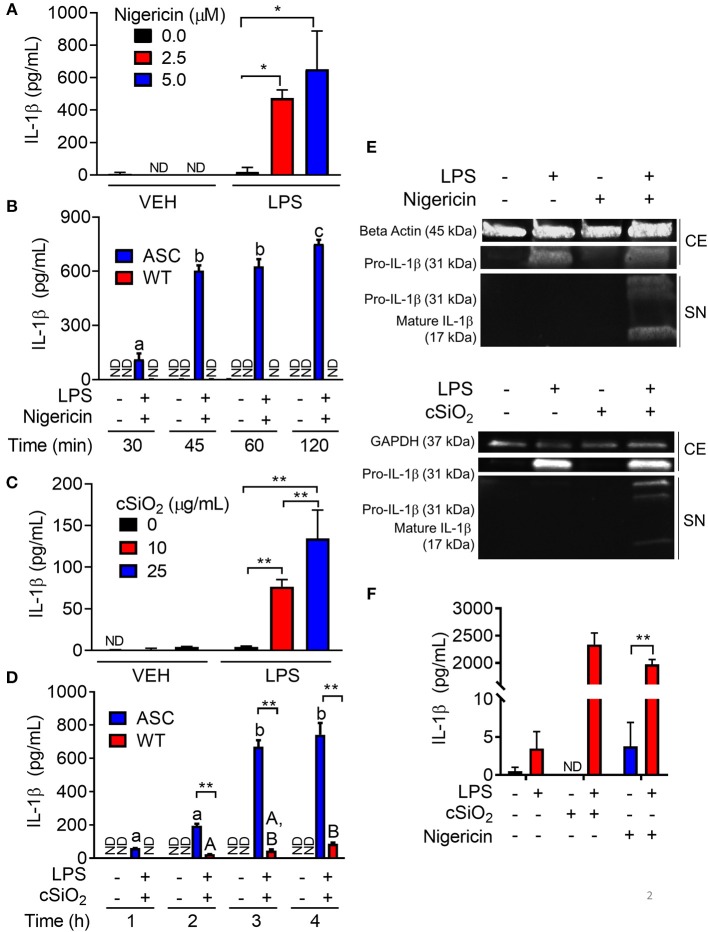
Nigericin- and cSiO_2_-induced IL-1β release are LPS- and ASC-dependent in RAW MΦs. **(A,C)** RAW-ASC cells were pretreated with 20 ng/ml LPS for 2 h, incubated with 0, 2.5, or 5.0 μM nigericin for 45 min **(A)**, or 0, 10, or 25 μg/ml cSiO_2_ for 4 h **(C)**, and then release of IL-1β measured. **(B,D)** RAW-ASC and RAW-WT were pretreated with VEH or LPS for 2 h, incubated with VEH or 10 μM nigericin **(B)** or 25 μg/ml cSiO_2_ **(D)**. IL-1β release was assessed at the indicated times. **(E)** Pro-IL-1β was present in the cell extracts (CE) of RAW-ASC MΦs treated with LPS, but only secreted into the supernatant (SN) with nigericin or cSiO_2_ treatment. IL-1β in the supernatant contained both the precursor and cleaved forms. **(F)** Bone marrow-derived macrophages were pretreated with VEH or LPS (20 ng/ml) for 2 h, incubated with VEH or 5 μM nigericin or 25 μg/ml cSiO_2_ and IL-1β release then assessed at 45 min or 4 h, respectively. Data presented as mean ± SEM, *n* = 3. ND = not detectable. Asterisks indicate significant differences between cell type **(B,D)** or treatment group **(A,C,E)** (*p < 0.05, **p < 0.01). Different letters indicate significant differences between treatment groups within each cell type **(B,D)** (*p* < 0.05). ELISA data are representative of three independent experiments. Western blots are representative of two independent experiments.

To confirm the efficacy of ASC transfection in conferring a functional inflammasome, the effects of the K^+^ ionophore nigericin, a prototypical activator of the NLRP3 inflammasome, were compared in RAW-ASC and RAW-WT cells. Nigericin elicited marked IL-1β secretion in LPS-primed RAW-ASC cells, whereas unprimed RAW-ASC cells were unresponsive (Figure 2A). Robust IL-1β release was evident in LPS-primed RAW-ASC cells as early as 30 min after nigericin treatment (Figure 2B). IL-1β release from LPS-primed RAW-WT cells was negligible at all time points, verifying that they lacked inflammasome activity. As found with nigericin, cSiO_2_ induced abundant IL-1β release in LPS-primed RAW-ASC cells within 1 h but not in RAW-WT or in unprimed RAW-ASC cells (Figures 2C,D). In response to both nigericin and cSiO_2_, release of IL-1β by RAW-ASC cells included both the inactive precursor and the bioactive mature form (Figure 2E). Collectively, IL-1β release in response to either activating stimulus was dependent on the presence of a priming signal and a functional inflammasome. Under identical experimental conditions, primary BMDM likewise released IL-1β in an LPS-dependent manner in response to nigericin and cSiO_2_ (Figure 2F) suggesting the RAW-ASC model was a relevant surrogate to investigate inflammasome activation in the macrophage.

### cSiO_2_-Induced Caspase-1 Activation Is LPS- and ASC-Dependent

**Figure 3:**
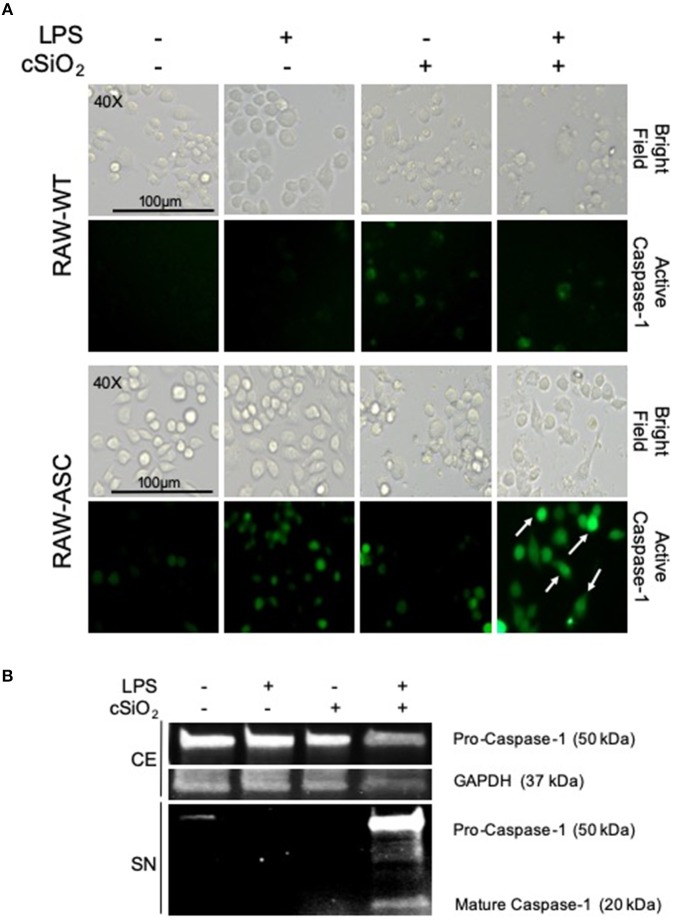
cSiO_2_-induced caspase-1 activation in RAW MΦs is LPS- and ASC-dependent. **(A)** RAW-WT and RAW-ASC cells were pretreated with VEH or 20 ng/ml LPS for 2 h and then incubated with VEH or 25 μg/ml cSiO_2_ for 4 h. Caspase-1 activation was assessed using fluorescent inhibitor probe FAM-YVAD-FMK. Treatment with LPS and cSiO_2_ induced minimal activation of caspase-1 in RAW-WT cells while RAW-ASC cells show robust activation of caspase-1 as indicated by green fluorescence (white arrows). Cells were imaged using an EVOS FL Auto Cell Imaging System; images representative of three independent experiments. **(B)** Pro-caspase-1 was constitutively expressed in RAW-ASC MΦs and detectable in the cell extract (CE). Pro-caspase-1 and cleaved, active caspase-1 (p20) were only present in the supernatant (SN) of cells treated with both LPS and cSiO_2_; Western blots are representative of two independent experiments.

During NLRP3 inflammasome activation, caspase-1 is post-translationally modified by cleavage to its mature, active form. FAM-YVAD-FMK, a fluorescent dye that binds intracellularly to cleaved, active caspase-1, was used to compare cSiO_2_-induced caspase-1 activation in RAW-ASC and RAW-WT cells. Caspase-1 was activated by cSiO_2_ only in LPS-primed RAW-ASC cells, confirming inflammasome-dependent activation (Figure 3A). These results were confirmed by Western blot analysis of cleaved caspase-1 in the media supernatant in RAW-ASC cells treated with both LPS and cSiO_2_ (Figure 3B). Consistent with these results, we observed the formation of ASC specks following LPS priming and cSiO_2_ or nigericin treatment (Figure S1A).

### Nigericin- and cSiO_2_- Induced IL-1α Release Differ With Regard to LPS- and ASC-Dependence

**Figure 4:**
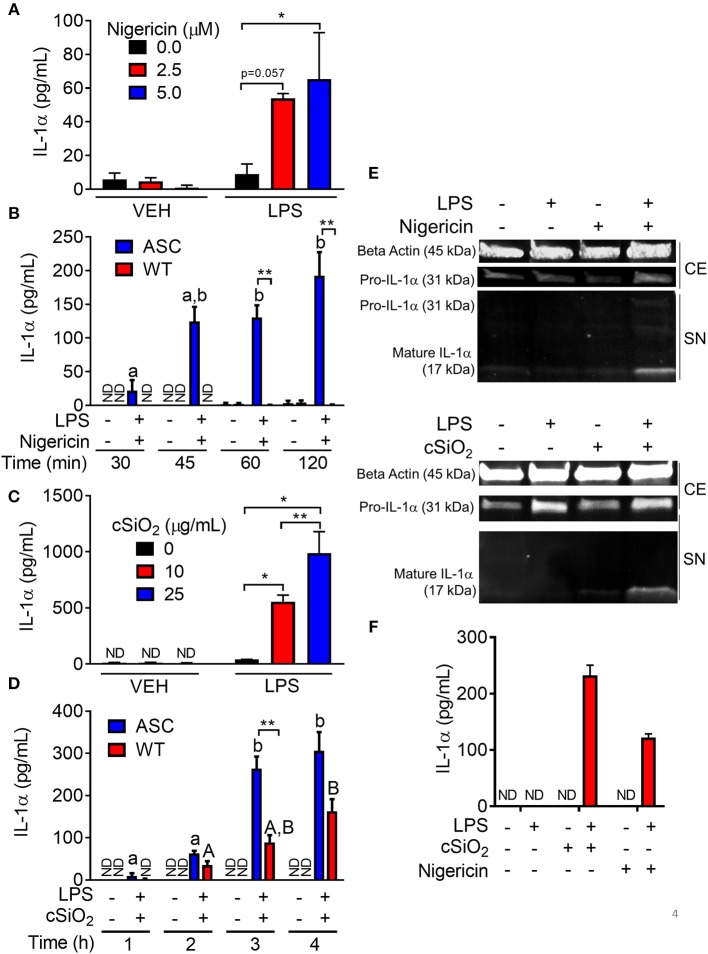
Nigericin- and cSiO_2_-induced IL-1α release is LPS- and ASC-dependent in RAW MΦs. **(A,D)** RAW-ASC cultures were pretreated with VEH or 20 ng/ml LPS for 2 h, incubated with 0, 2.5, or 5.0 μM nigericin for 45 min **(A)**, or 0, 10, or 25 μg/ml cSiO_2_ for 4 h **(C)**, and then release of IL-1α measured. **(B,D)** RAW-ASC and RAW-WT were pretreated with VEH or LPS for 2 h, incubated with VEH, 10 μM nigericin **(B)**, or 25 μg/ml cSiO_2_ **(D)** and release of IL-1α measured at the times indicated. **(E)** Pro-IL-1α was constitutively expressed and slightly upregulated in cell extracts (CE) of RAW-ASC MΦs treated with LPS, but only secreted into the supernatant (SN) with 5 μM nigericin or 25 μg/ml cSiO_2_ treatment. Both pro-IL-1α and mature IL-1α were detected in the supernatant. To detect IL-1α in the supernatant, LPS priming was extended to 5 h and protein concentrated 10x by methanol-chloroform precipitation. **(F)** Bone marrow-derived macrophages were pretreated with VEH or LPS (20 ng/ml) for 2 h, incubated with VEH or 5 μM nigericin or 25 μg/ml cSiO_2_ and IL-1α release then assessed at 45 min or 4 h, respectively. Data presented as mean ± SEM, *n* = 3. ND = not detectable. Asterisks indicate significant differences between cell type **(B,D)** or treatment group **(A,C,E)** (*p < 0.05, **p < 0.01). Different letters indicate significant differences between treatment groups within each cell type **(B,D)** (*p* < 0.05). ELISA data are representative of three independent experiments. Western blots are representative of two independent experiments.

Nigericin evoked a robust IL-1α response in LPS-primed RAW-ASC cells, but not unprimed cells (Figure 4A). The IL-1α response was detectable in supernatants of LPS-primed RAW-ASC cells within 30 min of nigericin addition but was not evident in LPS-primed RAW-WT supernatants up to 120 min following nigericin treatment (Figure 4B).

cSiO_2_-induced IL-1α release in RAW-ASC cells also required LPS pretreatment (Figure 4C). In contrast to nigericin findings, IL-1α concentrations in culture supernatants of cSiO_2_-treated RAW-WT cells were 30–50 percent of that observed in cSiO_2_-treated RAW-ASC cells (Figure 4D). These results suggest that cSiO_2_-induced release of some IL-1α occurs via mechanisms that do not involve NLRP3 inflammasome activation. IL-1α detected in the media of RAW-ASC cells was primarily the 17 kDa mature form (Figure 4E). Finally, as found in RAW-ASC cells, primary BMDM released IL-1α in response to nigericin and cSiO_2_ in an LPS-dependent manner (Figure 4F).

### Nigericin- but Not cSiO_2_-Induced Cell Death Is Inflammasome-Dependent

**Figure 5:**
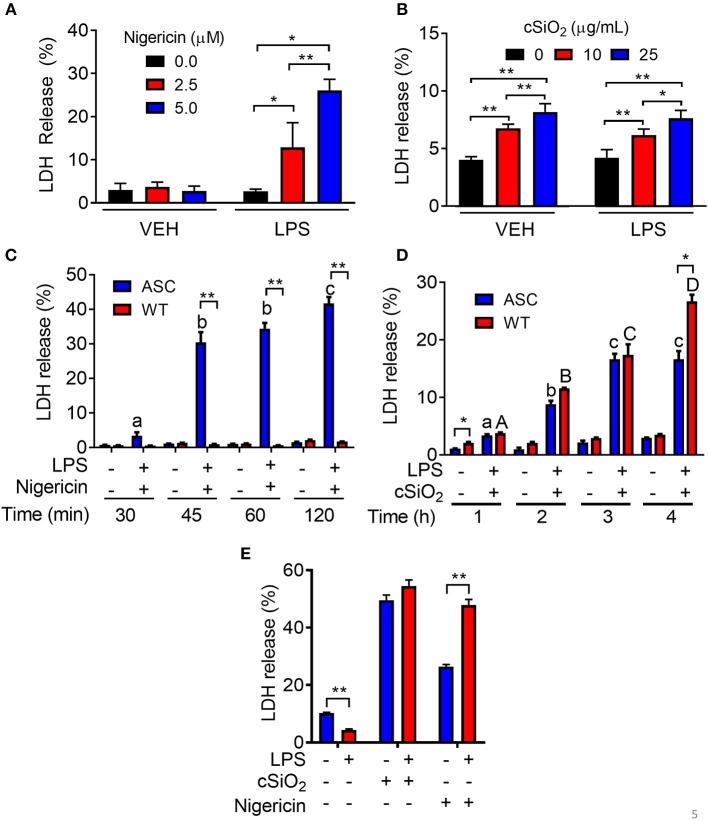
Nigericin-induced cell death is LPS- and ASC-dependent while cSiO_2_-induced cell death is LPS- and ASC-independent in RAW MΦs. **(A,C)** RAW-ASC cultures were pretreated with VEH or 20 ng/ml LPS for 2 h, incubated with 0, 2.5, or 5.0 μM nigericin for 45 min **(A)** or 0, 10, or 25 μg/ml cSiO_2_ for 4 h **(C)**, and then LDH measured. **(B,D)** RAW-ASC and RAW-WT MΦs were pretreated with VEH or 20 ng/ml LPS for 2 h, incubated with VEH, 10 μM nigericin **(B)**, or 25 μM cSiO_2_ **(D)** and then LDH release assessed at the indicated times. **(E)** Bone marrow-derived macrophages were pretreated with VEH or LPS (20 ng/ml) for 2 h, incubated with VEH or 5 μM nigericin or 25 μg/ml cSiO_2_ and then LDH release assessed at 45 min or 4 h, respectively. Data presented as mean ± SEM, *n* = 3. ND = not detectable. Asterisks indicate significant differences between cell type **(B,D)** or treatment group **(A,C,E)** (*p < 0.05, **p < 0.01). Different letters indicate significant differences between treatment groups within each cell type **(B,D)** (*p* < 0.05). Representative of three independent experiments.

Nigericin treatment induced LDH release in LPS-primed RAW-ASC cells but not in unprimed ones (Figure 5A). Concordant with these findings, nigericin elicited LDH release in RAW-ASC but not RAW-WT cells following LPS priming (Figure 5B) suggesting that death was inflammasome-dependent and thus consistent with pyroptosis. Unlike nigericin, cSiO_2_ induced LDH release in both LPS-primed and unprimed RAW-ASC cells (Figure 5C) and was largely equivalent in RAW-ASC and RAW-WT cells (Figure 5D), strongly indicating that cSiO_2_-induced cell death is not strictly inflammasome dependent and pyroptotic. Consistent with our findings in RAW-ASC cells, nigericin-induced cell death in BMDM was LPS-dependent whereas cSiO_2_-induced cell death was LPS-independent (Figure 5E).

### DHA Is Efficiently Incorporated Into RAW-ASC Cell Phospholipids

**Figure 6:**
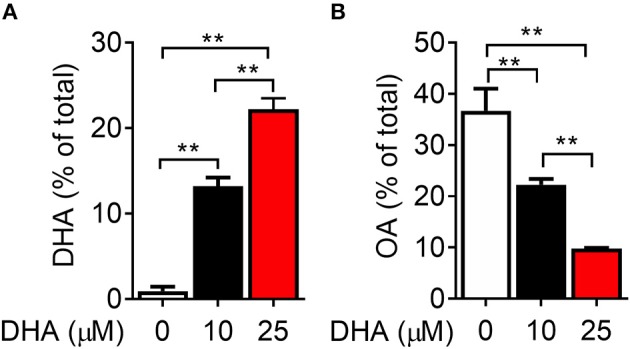
DHA is incorporated into RAW-ASC MΦ phospholipids at the expense of oleic acid (OA). RAW-ASC cells were incubated in serum-deprived media containing DHA-BSA complexes or BSA vehicle for 24 h. Cell phospholipids were extracted and analyzed for fatty acid content by GLC. In non-supplemented conditions, the major unsaturated fatty acid in the cell membrane is oleic acid (OA), which is the primary fatty acid component of fetal bovine serum. Incubation with DHA increased membrane content of DHA **(A)** while decreasing the membrane content of OA **(B)**. See Table 1 for complete fatty acid profile. Data presented as mean ± SEM, *n* = 3. Asterisks indicate significant difference (**p < 0.01). Representative of two independent experiments.

Following 24 h pre-incubation with DHA, delivered as a complex with BSA, the fatty acid was dose-dependently incorporated into the phospholipid fraction of RAW-ASC cells (Figure 6A). This occurred largely at the expense of oleic acid (OA) (Figure 6B), the major unsaturated fatty acid in the fetal bovine serum present in the culture medium (41). Along with DHA incorporation, there were significant decreases in ω-9 palmitoleic acid and ω-6 eicosadienoic acid and a significant increase in ω-3 eicosapentaenoic acid, which can be formed by enzymatic retroconversion of DHA (Table 1).

**Table 1:** DHA supplementation modulates phospholipid profile of RAW-ASC cells.

**Fatty acid**	**Common name**	**DHA (μM)**		
		**0**	**10**	**25**
C14:0	Myristic acid	2.94 ± 0.29	3.27 ± 0.28	4.90 ± 0.84^**^
C16:0	Palmitic acid	31.15 ± 3.10	31.34 ± 0.79	38.59 ± 1.25^**^
C16:1 ω-9	Palmitoleic acid	4.67 ± 1.04	3.13 ± 0.25	2.29 ± 0.50^**^
C18:0	Stearic acid	15.60 ± 9.05	22.03 ± 0.47	18.01 ± 1.21
C18:1 ω-9	Oleic acid	36.68 ± 4.32	22.23 ± 1.25^**^	9.77 ± 0.34^**^
C18:2 ω-6	Linoleic acid	0.63 ± 0.88	1.26 ± 0.11	0.78 ± 0.71
C20:2 ω-6	Eicosadienoic acid	4.16 ± 0.92	1.13 ± 0.68^**^	0.00 ± 0.00^**^
C20:4 ω-6	Arachidonic acid	3.27 ± 0.67	2.30 ± 0.47	1.80 ± 1.15
C20:5 ω-3	Eicosapentaenoic acid	0.00 ± 0.00	0.00 ± 0.00	1.13 ± 0.71
C22:4 ω-6	Adrenic acid	0.00 ± 0.00	0.15 ± 0.33	0.35 ± 0.79
C22:5 ω-3	ω3 docosapentaenoic acid	0.00 ± 0.00	0.17 ± 0.38	0.18 ± 0.40
C22:5 ω-6	ω6 docosapentaenoic acid	0.00 ± 0.00	0.14 ± 0.31	0.00 ± 0.00
C22:6 ω-3	Docosahexaenoic acid	0.91 ± 0.53	12.85 ± 0.49^**^	22.20 ± 1.42^**^
Total SFA		49.70 ± 5.81	55.44 ± 2.42^*^	60.63 ± 1.06^**^
Total MUFA		41.35 ± 5.29	25.05 ± 0.87^**^	11.88 ± 0.56^**^
Total ω-6		8.05 ± 1.02	5.77 ± 0.90^*^	3.71 ± 1.04^**^
Total ω-6		0.91 ± 0.53	13.73 ± 1.47^**^	23.77 ± 1.07^**^

Data shown are mean percent of total FA ± SEM (n = 6 per group) as determined by GLC. FA levels significantly different from control (0 μM DHA) represented by asterisks

(*p <0.05,

**p <0.01).

### DHA Inhibits Nigericin-Induced IL-1 Cytokine Release and Cell Death

**Figure 7:**
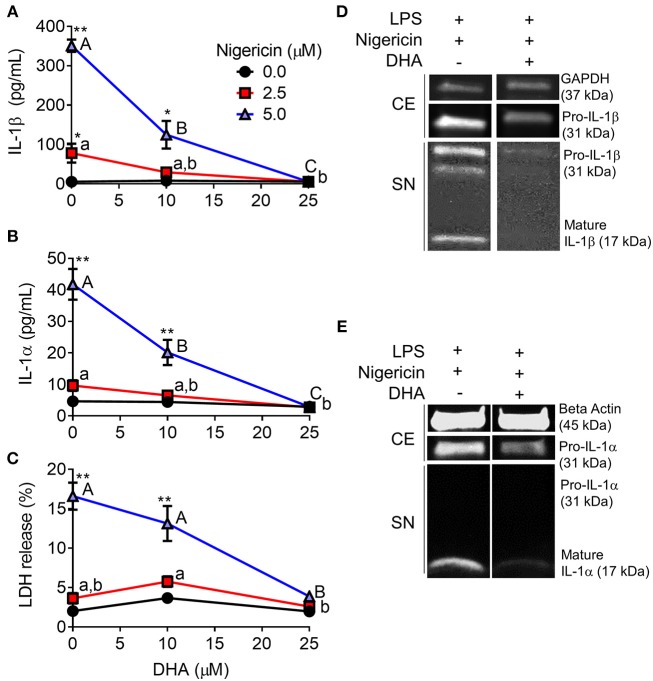
DHA supplementation suppresses nigericin-induced IL-1β and IL-1α release and cell death in RAW-ASC MΦs. RAW-ASC cells were incubated in serum-deprived RPMI containing DHA (0, 10, or 25 μM) or VEH (BSA) for 24 h. Cells were pretreated with 20 ng/ml LPS for 2 h, incubated with 0, 2.5, or 5.0 μM nigericin for 45 min, and then release of **(A)** IL-1β, **(B)** IL-1α, and **(C)** LDH measured. The presence of mature **(D)** IL-1β and **(E)** IL-1α in the supernatant were determined by Western blotting. For Western blots of IL-1α, LPS priming was extended to 5 h and supernatant concentrated 10x by methanol-chloroform precipitation. Data presented as mean ± SEM, *n* = 3. Significant differences between DHA-supplemented groups represented as different letters (uppercase for 5 μM group, lowercase for 2.5 μM group). Significant differences from vehicle control at each DHA concentration represented by asterisks (*p < 0.05, **p < 0.01). ELISAs and LDH assay are representative of three independent experiments. Western blots are representative of two independent experiments.

When RAW-ASC cells were pretreated with DHA and then primed with LPS, nigericin-induced release of IL-1β (Figure 7A) and IL-1α (Figure 7B) were suppressed by the ω-3 fatty acid in a concentration-dependent manner. DHA's effects corresponded to decreased intracellular pro-IL-1β and pro-IL-1α, as well as diminished extracellular mature IL-1β and IL-1α (Figures 7D,E). Finally, DHA pretreatment blocked nigericin-induced LDH release (Figure 7C), suggesting that DHA inhibits pyroptotic cell death.

### DHA Suppresses cSiO_2_-Induced IL-1 Cytokine Release and Caspase-1 Activation but Not Cell Death

**Figure 8:**
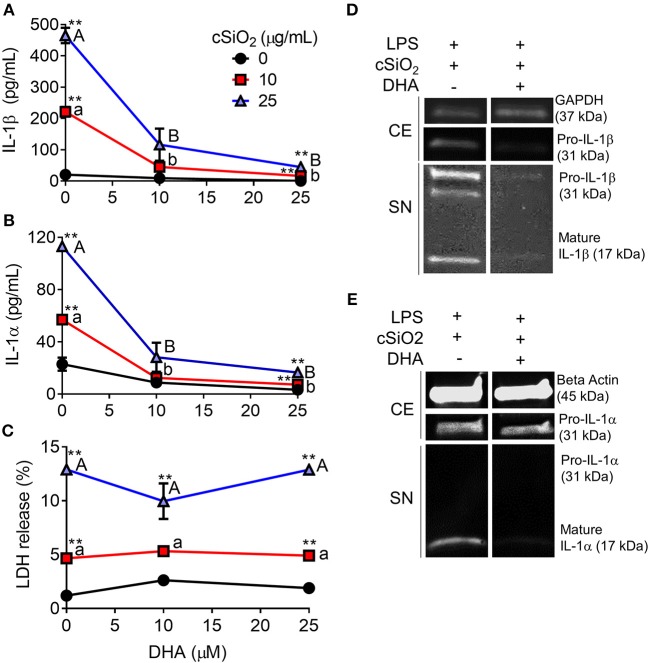
DHA inhibits cSiO_2_-induced IL-1β and IL-1α release but not cell death in RAW-ASC MΦs. RAW-ASC cells were incubated in serum-deprived RPMI containing DHA (0, 10, or 25 μM) or VEH (BSA) for 24 h. Cells were pretreated with 20 ng/ml LPS for 2 h, incubated with 0, 10, or 25 μg/ml cSiO_2_ for 4 h, and then release of **(A)** IL-1α, **(B)** IL-1β, and **(C)** LDH measured. The presence of mature **(D)** IL-1β and **(E)** IL-1α in the supernatant were determined by Western blotting. For Western blots of IL-1α, LPS priming was extended to 5 h and supernatant concentrated 10x by methanol-chloroform precipitation. Data presented as mean ± SEM, *n* = 3. Significant differences between DHA-supplemented groups represented as different letters (uppercase for 5 μM group, lowercase for 2.5 μM group). Significant differences from vehicle control at each DHA concentration represented by asterisks (**p < 0.01). ELISAs and LDH assay are representative of three independent experiments. Western blots are representative of two independent experiments.

**Figure 9:**
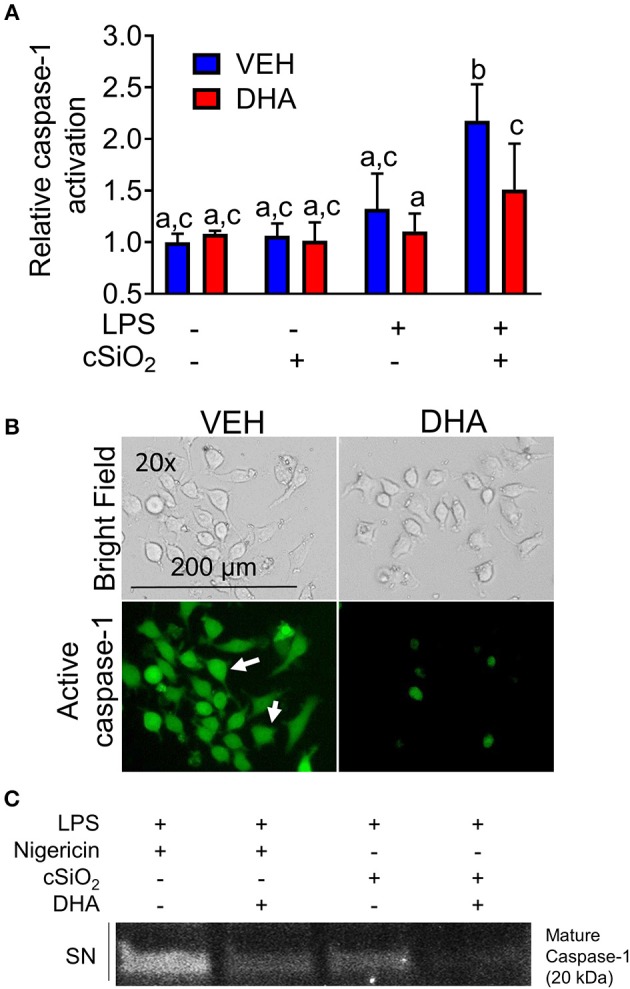
DHA supplementation suppresses cSiO_2_-induced caspase-1 activation in RAW-ASC MΦs. **(A)** RAW-ASC cells were incubated in serum-deprived RPMI containing DHA (25 μM) or VEH (BSA) for 24 h. Cultures were then primed with 20 ng/ml LPS for 2 h and incubated with or without 25 μg/ml cSiO_2_ for 4 h. Caspase-1 activation was assessed using fluorescent probe FAM-YVAD-FMK. A fluorescent microplate assay indicated that DHA suppressed cSiO_2_-induced caspase-1 activation. Fluorescence data pooled from two independent experiments and presented as mean ± SEM, *n* = 6. Significant differences between groups represented as different letters (*p* < 0.05). **(B)** Representative photomicrographs indicating that cells treated with LPS and cSiO_2_ showed robust activation of caspase 1 as indicated by green fluorescence (white arrows) and this was attenuated by supplementation with DHA. Cells were imaged using an EVOS FL Auto Cell Imaging System. Images are representative of three independent experiments. **(C)** Western blot analysis confirmed that DHA suppressed both the cleavage and release of active caspase-1 (p20) into the supernatant. Western blot data are representative of two independent experiments.

DHA concentration-dependently suppressed cSiO_2_-induced release of both IL-1β (Figure 8A) and IL-1α (Figure 8B). Likewise, DHA inhibited cSiO_2_-induced caspase-1 activation (Figure 9) and ASC speck formation (Figure S1B). DHA's inhibitory effects corresponded to reduced levels of intracellular pro-IL-1β and pro-IL-1α and extracellular mature IL-1β and IL-1α (Figures 8D,E). However, DHA did not affect cell death induced by cSiO_2_ (Figure 8C), further suggesting that cSiO_2_-induced cell death did not involve inflammasome activation and pyroptosis.

### DHA Suppresses IL-1 Cytokine Release Triggered by Alum and MSU Crystals

**Figure 10:**
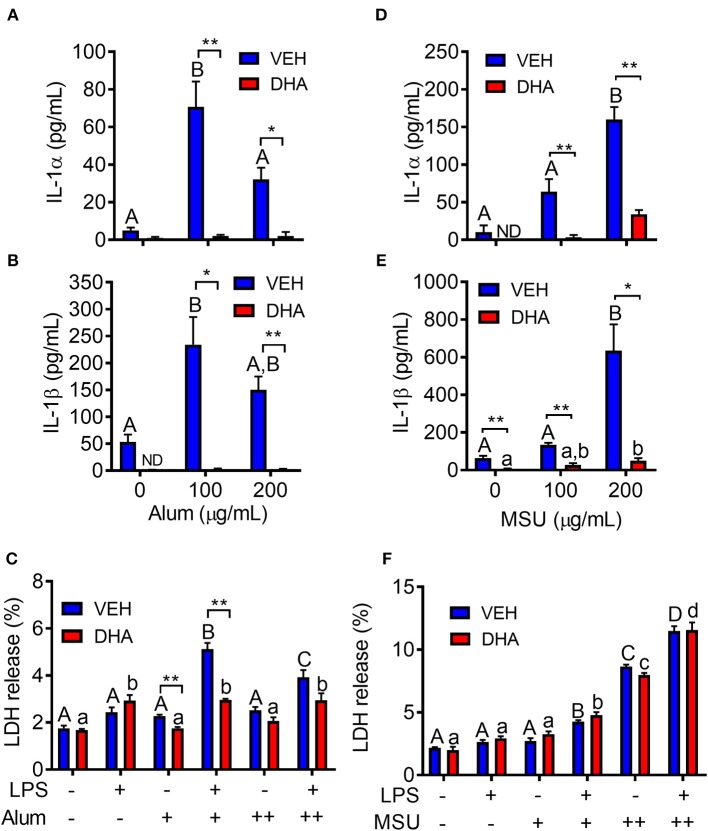
Alum- and MSU crystal-induced IL-1 cytokine release is suppressed by DHA. RAW-ASC cells were incubated in serum-deprived RPMI containing DHA (25 μM) complexed with BSA or VEH (BSA only) for 24 h. Cells were pretreated with 20 ng/ml LPS for 2 h, incubated with 0 (-), 100 (+), or 200 (++) μg/ml alum **(A-C)** or MSU **(D–F)** for 8 h, and then release of IL-1α, **(A,D)**, IL-1β **(B,E)**, and LDH **(C,F)** measured. Data presented as mean ± SEM, *n* = 3. Asterisks indicate significant differences between DHA and BSA treated cells (*p < 0.05, **p < 0.01). Different letters indicate significant differences between treatment groups within each VEH treated cells (uppercase letters) or DHA treated cells (lowercase letters) (*p* < 0.05). Data representative of three independent experiments.

Following priming with LPS, both alum (Figures 10A,B) and MSU (Figures 10D,E) induced robust release of both IL-1α and IL-1β. In both instances, release of IL-1 cytokines was ablated by supplementation with DHA. Unlike cSiO_2_-induced LDH release, responses to alum and MSU were extremely modest, slightly potentiated by LPS priming, and negligibly affected by DHA (Figures 10C,F).

### DHA Interferes With LPS Priming by Activating PPARγ

**Figure 11:**
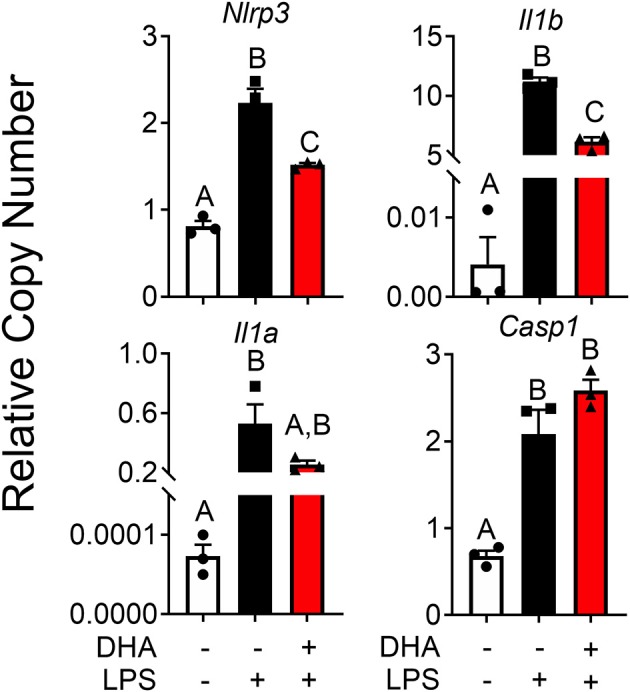
DHA supplementation downregulates LPS-induced inflammasome gene expression and intracellular IL-1 cytokines in RAW-ASC cells. RAW-ASC cells were incubated in serum-deprived RPMI containing DHA (25 μM) or VEH (BSA) for 24 h. Cultures were then incubated with 20 ng/ml LPS for 6 h. Assessment of mRNA levels by qRT-PCR showed that LPS priming induced *Nlrp3, Casp1, Il1b*, and *Il1a* mRNA expression and upregulation of *Nlrp3, Il1b*, and *Il1a* was suppressed by DHA. Gene expression represented as copy number relative to *Gapdh*. Data presented as mean ± SEM, *n* = 3. Letters indicate significant differences between groups (p<0.05). Data are representative of two independent experiments.

**Figure 12:**
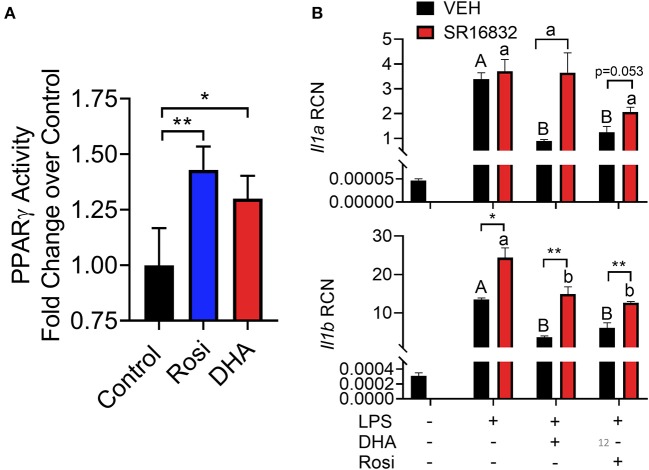
**(A)** A TransAM™ PPARγ transcription factor assay was used to assess PPARγ activity in nuclear extracts of DHA (10 μM) and PPARγ agonist rosiglitazone (10 μM) treated RAW-ASC cells. Data presented as mean ± SEM, *n* = 2. Asterisks indicate significant relative to the control (*p < 0.05, **p < 0.01). **(B)** RAW-ASC cells were incubated in serum-deprived RPMI containing DHA (10 μM), rosiglitazone (10 μM), PPARγ antagonist SR16832 (100 nM), or VEH (BSA) for 24 h. Cultures were then incubated with 20 ng/ml LPS for 3.5 h. Assessment of mRNA levels by qRT-PCR showed that the PPARγ antagonist SR16832 blocked DHA and rosiglitazone-dependent suppression of LPS-induced gene expression. Gene expression represented as copy number relative to *Gapdh*. Asterisks indicate significant differences between DHA and BSA treated cells (*p < 0.05, **p < 0.01). Data presented as mean ± SEM, *n* = 3. Different letters indicate significant differences between treatment groups within VEH treated cells (uppercase letters) or SR16832 treated cells (lowercase letters) (*p* < 0.05). Representative of two independent experiments.

DHA pretreatment significantly suppressed LPS-induced expression of *Nlrp3* and *Il1b* (Figure 11). A similar trend (*p* = 0.100) of DHA inhibition was observed for LPS-induced *Il1a* mRNA expression. DHA potentially inhibits IL-1 cytokine and NLRP3 transcription by activating PPARγ, a well-known transrepressor of NF-κB (42). To test this possibility, PPARγ binding activity was measured in nuclear extracts of RAW-ASC cells treated with DHA or with the PPARγ agonist rosiglitazone as a positive control. Significantly more active PPARγ was detectable in nuclear extracts from the rosiglitazone- and DHA-treated cells than those from vehicle-treated cells (Figure 12A). Consistent with these findings, both rosiglitazone- and DHA-mediated suppression of IL-1 cytokine gene expression was suppressed in cells treated with the PPARγ antagonist SR16832 (Figure 12B). Although LPS treatment induced phosphorylation of IKKα/β, degradation of IκBα, NF-κB phosphorylation, and nuclear translocation of NF-κB (Figure S2), none of these effects were influenced by DHA.

## Discussion

**Figure 13:**
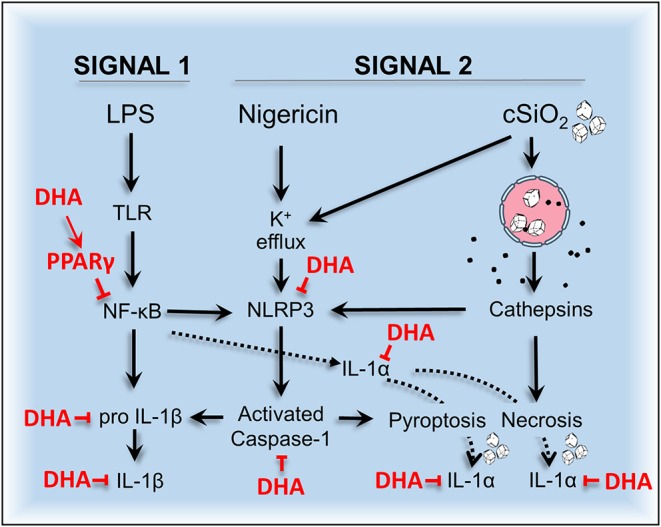
Putative model for the protective effects of DHA against nigericin- and cSiO_2_-induced inflammasome activation, IL-1 cytokine release, and death in MΦs. DHA inhibits nigericin- and cSiO_2_-induced inflammasome activation as measured by IL-1β maturation and release and caspase-1 activation. DHA also suppresses nigericin- and cSiO_2_-induced IL-1α cleavage and release and cell death. Cell death is wholly suppressed by DHA in nigericin-treated MΦs but only partially suppressed in cSiO_2_-treated MΦs. Collectively, these inhibitory effects are linked to suppression of genes (*Nlrp3, Il1b*, and *Il1a*) regulated by the transcription factor NF-κB.

Dysregulation of inflammasomes has been implicated as a contributing factor in lupus and other autoimmune diseases (43). Airway exposure to cSiO_2_ triggers prolific inflammation in the lung and onset of localized and systemic autoimmunity in lupus-prone mice (2), but inclusion of DHA in the diet abrogates these effects (3, 4, 44). The early mechanisms for DHA's ameliorative actions are as yet unclear. To address this knowledge gap, we tested the hypothesis that DHA suppresses cSiO_2_-induced NLRP3 inflammasome activation, IL-1 cytokine release, and cell death in the macrophage. Like BMDM, RAW-ASC cells were found to be capable of robust NLRP3 inflammasome activation, and therefore suitable surrogates to investigate DHA's effects on macrophage responses to cSiO_2_. We report for the first time that DHA at physiologically relevant concentrations interferes with cSiO_2_-induced inflammasome activation and release of mature IL-1α and IL-1β but not with cell death. As depicted in Figure 13, DHA likely acts at the level of priming (i.e., Signal 1), as evidenced by its suppression of LPS-induced *Nlrp3, Il1b*, and *Il1a* gene expression that influenced later responses to cSiO_2_ or nigericin (Signal 2). Importantly, suppression by DHA was linked to increased PPARγ activity.

An early and critical response to airborne cSiO_2_ exposure is robust release of IL-1 cytokines by AMΦs (16) Thus, it is noteworthy that DHA suppressed release of both mature IL-1α and IL-1β following treatment with cSiO_2_ and other inflammasome activators (MSU, alum, and the canonical inflammasome inducer nigericin). Although IL-1α and IL-1β share many characteristics, there are distinctions in the mechanisms by which they are expressed, processed, and released. Pro-IL-1α is constitutively expressed in many cell types, including immune cells where it can be further upregulated by physiological stimuli, including oxidative stress, hormonal stimulation, and exposure to cytokines (including IL-1β and IL-1α itself) (15). Pro-IL-1β is primarily expressed by immune cells and is rapidly induced by inflammatory stimuli. Both exist as 31 kDa precursors that can be cleaved to 17–18 kDa mature forms (45). IL-1α is bioactive in both the precursor and mature forms, however, it has been reported that IL-1α activity is enhanced upon cleavage by calpains, which may be activated by cSiO_2_-induced Ca^2+^ influx (46). IL-1β is only active in its mature form and can be cleaved by caspase-1 during inflammasome activation. IL-1β may also be cleaved in a caspase-independent manner by proteinases produced by other immune cell types (47). Additionally, both pro-IL-1α and pro-IL-1β released from dying cells can be processed by extracellular proteases (45).

Unlike most cytokines, IL-1 cytokines lack secretory sequences targeting them to the endoplasmic reticulum and Golgi apparatus for processing and release from the cell. Rather, mature IL-1β has been shown to be released through pores formed by gasdermin D (GSDMD) (48–50). Though it has not been confirmed experimentally, it is conceivable that mature IL-1α may also be released in this manner. During inflammasome activation, GSDMD is cleaved by caspase-1, whereupon the N-terminal fragment localizes to the cell membrane and oligomerizes to form pores (9). A further feature of GSDMD pores is their capacity to collapse the plasma membrane, causing lytic pyroptotic cell death and releasing additional alarmins and cytokines that act as priming signals for the NLRP3 inflammasome (9, 16, 47).

Since cSiO_2_ clearance from the lung is very slow (51), the persistent presence of this particle elicits repeated cycles in AMΦs involving phagocytosis of free cSiO_2_ → phagolysosome permeabilization → death → release of cell autoantigens and reemergence of free cSiO_2_. Like cSiO_2_, other exogenous and endogenous crystals (e.g., alum and MSU, respectively) also evoke phagolysosome permeabilization (8, 52, 53). These crystals elicit pyroptosis (54) as well as inflammasome-independent cell death via apoptotic and necrotic pathways (55, 56). Our data here suggest that crystal-induced death in RAW-ASC cells was, to a large extent, inflammasome-independent. The release of IL-1α during other types of death associated with inhalation of crystalline substances (9, 56–58) is consistent with our observation of this cytokine in cell supernatant following cSiO_2_ treatment of LPS-treated RAW-WT cells.

Numerous preclinical and clinical studies show consuming long chain ω-3 PUFAs such as DHA and eicosapentaenoic acid (C20:5 ω-3; EPA) can reduce chronic inflammatory and autoimmune conditions (44, 59). Western diets tend to exclude these pro-resolving ω-3s and more typically contain high concentrations of proinflammatory ω-6 PUFAs like linoleic acid (C18:2 ω-6; LA) and arachidonic acid (C20:4 ω-6; ARA) found in plant- and animal-derived lipids. Americans consume many times more ω-6s than ω-3s, so tissue phospholipid fatty acids skew heavily toward ω-3 deficiency (60). Several marine algae proficiently catalyze formation of DHA and EPA. Oily fish (e.g., salmon, mackerel) and small crustaceans (e.g., krill) bioconcentrate ω-3s into their membrane phospholipids by consuming the marine microalgae (61). Individuals can increase DHA and EPA tissue incorporation and correct ω-3 deficiency by consuming fish or dietary supplements with fish oil, krill oil, or microalgal oil.

Following dietary supplementation, DHA concentrations in the lung and other tissues in the lupus-prone NZBWF1 mouse correlate with decreased cSiO_2_-triggered autoimmune pathogenesis (3, 4). Significantly, levels of *in vitro* DHA incorporation observed in the present study are similar to those found *in vivo* for mice fed diets supplemented with DHA, suggesting that *in vitro* concentrations of DHA (10 and 25 μM) used here are physiologically relevant. Our findings are consistent with prior reports that DHA suppresses NLRP3 inflammasome activation in other primary and transformed MΦ cell lines stimulated by nigericin (18, 62). The demonstration here that DHA suppresses expression of three NF-κB dependent genes is concordant with reports that activity of this transcription factor might be inhibited by ω-3 PUFAs, both *in vitro* and *in vivo* (59). Notably, our laboratory has previously shown that intranasally instilling mice with cSiO_2_ upregulates many NF-κB targets, including but not limited to MCP-1, TNFα, BAFF, and IL-6. The expression of these genes is significantly reduced in animals supplemented with dietary DHA, suggesting involvement of this pathway *in vivo* (3, 4, 63).

Our finding here that DHA activates PPARγ is consistent with other studies in RAW 264.7 cells (64, 65) and in other macrophage cell lines (42). PPARγ's capacity to interfere with NF-κB-dependent gene expression (66) might partially explain DHA interference with IL-1 and NLRP3 gene expression. The exact mechanism of PPARγ-dependent inhibition of NF-κB appears to depend on the gene being upregulated. We found here that DHA did not impede nuclear translocation of NF-κB. A similar scenario has been observed for *iNos* expression in RAW 264.7 cells. In that case, PPARγ-dependent suppression of *iNos* does not impact NF-κB binding but rather represses LPS-induced *iNos* expression by preventing the recruitment of the proteasome machinery required to clear co-repressors from the *iNos* promoter (67). In a separate study in RAW 264.7 cells, activated PPARγ interacts directly with NF-κB to prevent it from binding to the *Il12* promoter (68). Other studies in human colonic cells and mouse embryonic fibroblast studies reveal that PPARγ has E3 ligase activity, and can induce degradation of NF-κB (69). Still others show that PPARγ promotes nuclear export of NF-κB in Caco-2 cells (70).

We cannot exclude the possibility of other mechanisms besides PPARγ that might contribute to our findings (71, 72). For example, ω-3 PUFAs have been shown to affect the physical properties of the cell membrane. Both TLR4 and IL-1R1 activation require the oligomerization of multiple receptors, a process that requires structural alteration of the plasma membrane (73, 74). Increased phospholipid ω-3 PUFA content reduces the formation of lipid rafts, thus suppressing inflammatory signaling pathways that depend on clustering of transmembrane receptors (73–75). Alternatively, free DHA can be cleaved from the membrane and act as a ligand for anti-inflammatory receptors. *In vitro* studies reveal that DHA activates G-protein coupled receptors (GPCRs) FFAR1/GPR40 and FFAR4/GPR120 (18, 76). Previous studies indicate that activation of FFAR1/GPR40 and FFAR4/GPR120 by extracellular free DHA prevents TAB1 from binding TAK1, which is a necessary step in LPS-induced NF-κB activation (77). Finally, DHA-derived metabolites, many of which are termed specialized pro-resolving mediators (SPMs), are associated with the resolution of inflammation. Many reports of the bioactivity of SPMs support their potential to attenuate inflammasome activation (22, 78–82), potentially by binding to GPCRs involved in inhibiting NF-κB signaling (22, 81, 83–85). In general, these mechanisms culminate in inhibition of NF-κB, which we did not observe in our model. Accordingly, while outside the scope of this study, further clarification is needed to delineate the relative contributions of PPARγ-dependent and –independent mechanisms that might contribute to DHA-mediated suppression of IL-1 cytokine and NLRP3 gene expression.

Taken together, we have demonstrated that increasing the DHA content of membrane phospholipids suppresses cSiO_2_-induced inflammasome activation and release of IL-1 cytokines and that these effects are potentially linked to PPARγ activation and interference with NF-κB-driven gene expression (Figure 13). Understanding how DHA and other ω-3 PUFAs influence crystal-mediated pathogenesis could potentially lead to harnessing dietary modulation of the lipidome as an intervention against chronic inflammatory and autoimmune diseases involving the inflammasome.

## Data Availability

The raw data supporting the conclusions of this manuscript will be made available by the authors, without undue reservation, to any qualified researcher.

## Author Contributions

KW and JW: study design, data analyses/interpretation, and manuscript preparation. KG: data analysis/interpretation. LR: data analysis/interpretation and manuscript preparation. MB: optimization of RAW-ASC model and data analysis/interpretation. MG: data analysis/interpretation and generation of RAW-ASC model. AH: experimental design, data interpretation, manuscript writing, and project funding. JP: planning, coordination, oversight, manuscript preparation/submission, and project funding.

### Conflict of Interest Statement

The authors declare that the research was conducted in the absence of any commercial or financial relationships that could be construed as a potential conflict of interest.

## Supplementary Material

Figure S1 Visualization of ASC-CFP specks in RAW-ASC cells stimulated with LPS, nigericin and/or cSiO_2_. Cells were incubated in serum-deprived media containing VEH (BSA) **(A)** or 25 μM DHA **(B)** for 24 h. Cells were then primed for 2 h with 500 ng/mL LPS and treated with 5 μM nigericin (45 min) or 25 μg/mL cSiO_2_ (4 h). ASC specks (white arrows) were visualized using an EVOS FL Auto Cell Imaging System equipped with a CFP light cube. Photomicrographs representative of two independent experiments.

Figure S2 Assessment of NF-κB activation. RAW-ASC cells were incubated in serum-deprived media containing the indicated concentration DHA or VEH (BSA) for 24 h. Cells were then treated with 20 ng/mL LPS and collected at the indicated times. Cell lysates were fractionated to obtain separate cytoplasmic and nuclear extracts. **(A)** NF-κB translocation was assessed by the presence of NF-κB in nuclear extracts. PCNA was used as a loading control. **(B)** Canonical NF-κB signaling was assessed by IκBα degradation in cytoplasmic extracts. Beta actin was used as a loading control. **(C)** Cells were treated with ng/mL LPS for 30 min. In the cytoplasm, phosphorylation of IKKα/β and degradation of IκBα were measured to assess activation of the NF-κB signaling pathway. In the nucleus, the NF-κB p65 subunit was measured to evaluate nuclear translocation. Actin and PCNA were used as cytoplasmic and nuclear loading controls, respectively.

Table S1 List of products and reagents.
