# Supplementary figures and images for: Docosahexaenoic Acid Suppresses Silica-Induced Inflammasome Activation and IL-1 Cytokine Release by Interfering With Priming Signal
Source: Front Immunol. 2019 Sep 20;10:2130. doi: 10.3389/fimmu.2019.02130 (PMC6763728; doi:10.3389/fimmu.2019.02130)

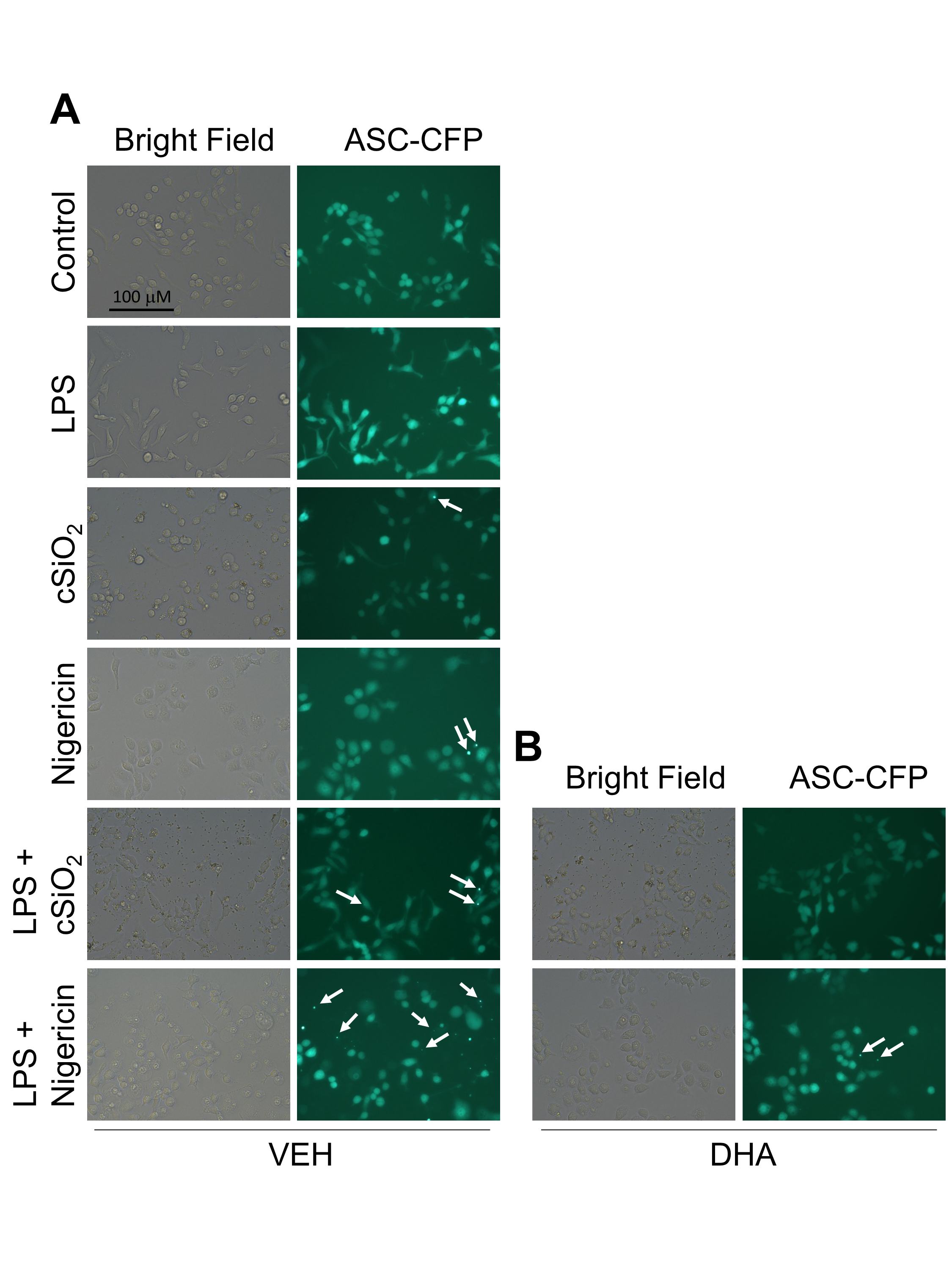

Supplement: Figure S1 — Visualization of ASC-CFP specks in RAW-ASC cells stimulated with LPS, nigericin and/or cSiO2. Cells were incubated in serum-deprived media containing VEH (BSA) (A) or 25 μM DHA (B) for 24 h. Cells were then primed for 2 h with 500 ng/mL LPS and treated with 5 μM nigericin (45 min) or 25 μg/mL cSiO2 (4 h). ASC specks (white arrows) were visualized using an EVOS FL Auto Cell Imaging System equipped with a CFP light cube. Photomicrographs representative of two independent experiments. [file Image_1.TIF]

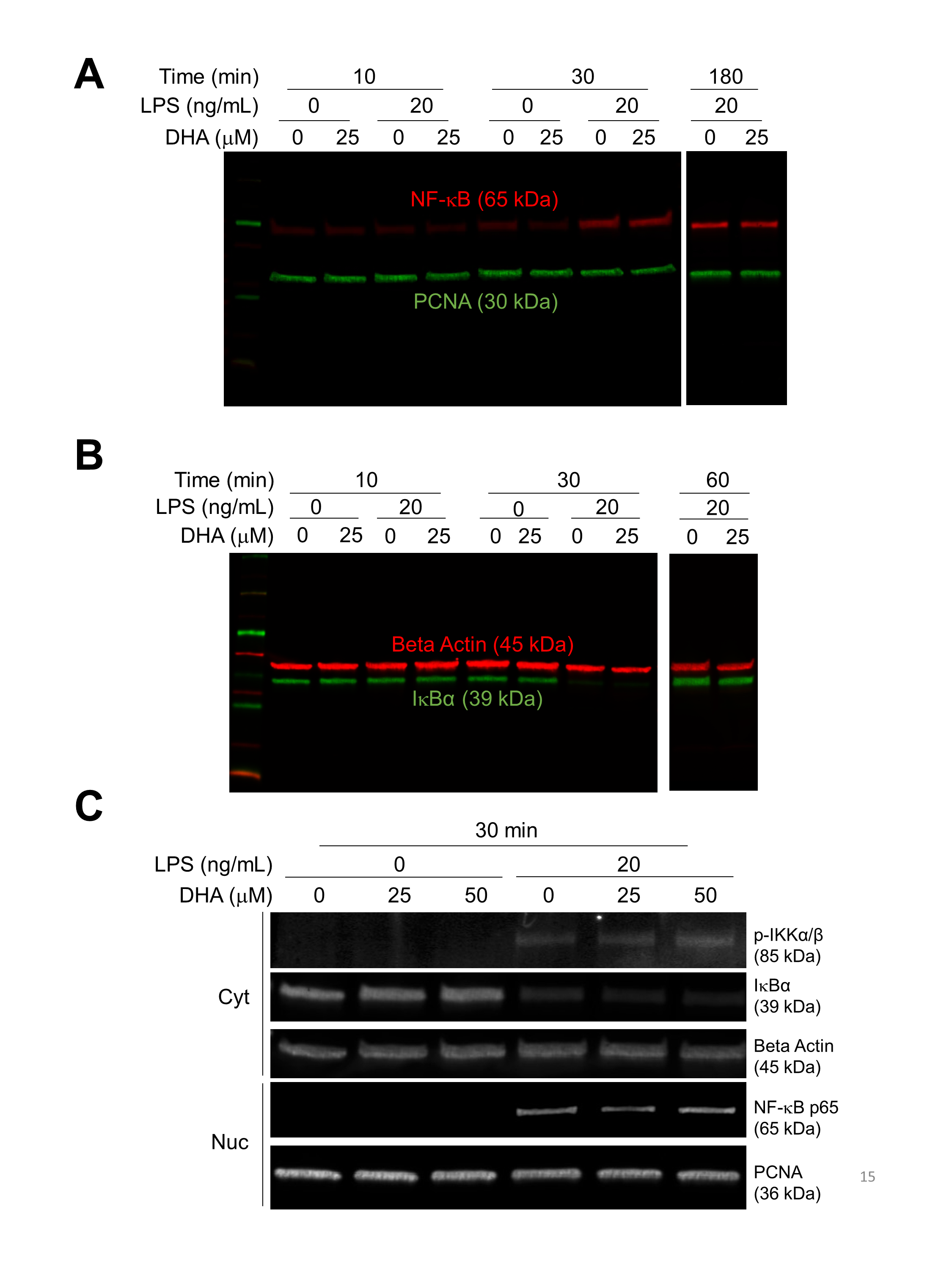

Supplement: Figure S2 — Assessment of NF-κB activation. RAW-ASC cells were incubated in serum-deprived media containing the indicated concentration DHA or VEH (BSA) for 24 h. Cells were then treated with 20 ng/mL LPS and collected at the indicated times. Cell lysates were fractionated to obtain separate cytoplasmic and nuclear extracts. (A) NF-κB translocation was assessed by the presence of NF-κB in nuclear extracts. PCNA was used as a loading control. (B) Canonical NF-κB signaling was assessed by IκBα degradation in cytoplasmic extracts. Beta actin was used as a loading control. (C) Cells were treated with ng/mL LPS for 30 min. In the cytoplasm, phosphorylation of IKKα/β and degradation of IκBα were measured to assess activation of the NF-κB signaling pathway. In the nucleus, the NF-κB p65 subunit was measured to evaluate nuclear translocation. Actin and PCNA were used as cytoplasmic and nuclear loading controls, respectively. [file Image_2.TIF]
