# Supplementary material for: Docosahexaenoic Acid Suppresses Silica-Induced Inflammasome Activation and IL-1 Cytokine Release by Interfering With Priming Signal
Source: Front Immunol. 2019 Sep 20;10:2130. doi: 10.3389/fimmu.2019.02130 (PMC6763728; doi:10.3389/fimmu.2019.02130)
Supplement: Table S1 — List of products and reagents. [file Data_Sheet_1.PDF]

Supplemental Table 1. List of products and reagents

Product	Company	City	Cat#
Lipofectamine 2000	Invitrogen Life Technologies	Carlsbad, CA	11668030
Blasticidin	Inivogen	San Diego, CA	ant-bl-05
Phenol red-free RPMI 1640	Gibco (Thermo Fisher Scientific)	Waltham, MA	11835030
Fetal Bovine Serum	Gibco (Thermo Fisher Scientific)	Waltham, MA	100099141
Penicillin Streptomycin	Invitrogen Life Technologies	Carlsbad, CA	15140122
RBC lysis buffer for mouse	Alfa Aesar (Thermo Fisher Scientific)	Waltham, MA	J62150
DMEM	Gibco (Thermo Fisher Scientific)	Waltham, MA	11965092
Silica	Pennsylvania Glass Sand Corp	Pittsburgh, PA	Min-U-Sil-5
Dulbecco's Phosphate Buffered Saline	Millipore Sigma	Burlington, MA	D8537
Monosodium Urate Crystals	Inivogen	San Diego, CA	tlrl-msu
Alum Crystals	Invivogen	San Diego, CA	tlrl-alk
Lipopolysaccharide from *Salmonella Enterica*	Millipore Sigma	Burlington, MA	L6143
Nigericin	Millipore Sigma	Burlington, MA	N7143
Fatty acid-free Bovine Serum Albumin (BSA)	Millipore Sigma	Burlington, MA	A8806 5G
Docosahexaenoic Acid	Cayman Chemical	Ann Arbor, MI	6217 545
IL-1β DuoSet ELISA	R&D Systems	Minneapolis, MN	DY401
IL-1α DuoSet ELISA	R&D Systems	Minneapolis, MN	DY400
Pierce™ TMB Substrate Kit	Thermo Fisher Scientific	Waltham, MA	34021
Bovine Serum Albumin	Millipore Sigma	Burlington, MA	A3912-100G
FAM-FLICA Caspase 1 Assay Kit	ImmunoChemistry Technologies	Bloomington, MN	97

Pierce BCA Protein Assay Kit	Thermo Fisher Scientific	Waltham, MA	23225
Triton X-100	Millipore Sigma	Burlington, MA	T8787
Nuclear Extract Kit	Active Motif	Carlsbad, CA	40010
TransAM® PPARγ Transcription Factor ELISA kit	Active Motif	Carlsbad, CA	40196
RNeasy Isolation Kit	Qiagen	Germantown, MD	74106
High Capacity RNA to cDNA RT Kit	Applied Biosystems (Thermo Fisher Scientific)	Waltham, MA	4387406
GAPDH Taqman Probe	Thermo Fisher Scientific	Waltham, MA	Mm99999915_g1
NLRP3 Taqman Probe	Thermo Fisher Scientific	Waltham, MA	Mm00840904_m1
Caspase-1 Taqman Probe	Thermo Fisher Scientific	Waltham, MA	Mm00438023_m1
IL-1β Taqman Probe	Thermo Fisher Scientific	Waltham, MA	Mm01336189_m1
IL-1α Taqman Probe	Thermo Fisher Scientific	Waltham, MA	Mm00439620_m1
TaqMan Fast Advanced Master Mix	Thermo Fisher Scientific	Waltham, MA	4444963
Halt Protease Inhibitor	Thermo Fisher Scientific	Waltham, MA	87785
NE-PER Nuclear and Cytoplasmic Extraction Reagents	Thermo Fisher Scientific	Waltham, MA	78833
4-20% Mini Protean Gel	Bio-Rad	Hercules, CA	4561094
10x TGS Running buffer	Bio-Rad	Hercules, CA	1610732
Trans-Blot® Turbo™ RTA Mini PVDF transfer kit	Bio-Rad	Hercules, CA	1704274
Gel transfer stacks (RTA Mini PVDF Transfer Kit)	Bio-Rad	Hercules, CA	1704272
TransBlot Turbo	Bio-Rad	Hercules, CA	1704510
iBind Flex Western Blot System	Thermo Fisher Scientific	Waltham, MA	SLF2000

Goat Anti-mouse IL-1β Antibody	R&D Systems	Minneapolis, MN	AF-401-NA
Rabbit Anti-mouse IL-1α Antibody	Cell Signaling Technology	Danvers, MA	50794
Mouse Anti-IκBα	Cell Signaling Technology	Danvers, MA	4814
Rabbit Anti-phospho-IKKα/β	Cell Signaling Technology	Danvers, MA	2697
Rabbit Anti-NF-κB p65	Cell Signaling Technology	Danvers, MA	8242
Rabbit Anti-GAPDH Antibody	Cell Signaling Technology	Danvers, MA	3907
Rabbit anti-beta-actin	Cell Signaling Technology	Danvers, MA	4970
Mouse anti-PCNA	Cell Signaling Technology	Danvers, MA	2586
Donkey Anti-goat IRDye 800CW	LI-COR Biotechnology	Lincoln, NE	926-32214
Goat Anti-rabbit IRDye 680RD	LI-COR Biotechnology	Lincoln, NE	925-68071
Goat Anti-Mouse IRDye 800CW	LI-COR Biotechnology	Lincoln, NE	925-32210
Heptadecanoic Acid Standard	NuChek Prep	Elysian, MN	N-17-A
Methanolic BF_3	Millipore Sigma	Burlington, MA	61626
Gas Chromatography Capillary Column	Agilent J&W	Santa Clara, CA	DB-23
Standard Mix	NuChek Prep	Elysian, MN	GLC682
